# Weighing the prognostic role of hyponatremia in hospitalized patients with metastatic solid tumors: the HYPNOSIS study

**DOI:** 10.1038/s41598-019-49601-3

**Published:** 2019-09-10

**Authors:** Giovanni Fucà, Luigi Mariani, Salvatore Lo Vullo, Giulia Galli, Rossana Berardi, Massimo Di Nicola, Claudio Vernieri, Daniele Morelli, Katia Dotti, Ilaria Fiordoliva, Silvia Rinaldi, Cecilia Gavazzi, Filippo Pietrantonio, Marco Platania, Filippo de Braud

**Affiliations:** 1 Medical Oncology Department, Fondazione IRCCS Istituto Nazionale dei Tumori di Milano, Milan, Italy; 2 Clinical Epidemiology and Trials Organization Unit, Fondazione IRCCS Istituto Nazionale dei Tumori di Milano, Milan, Italy; 3 Clinica Oncologica, Università Politecnica delle Marche - AOU Ospedali Riuniti di Ancona, Ancona, Italy; 4 Pathology and Laboratory Medicine Department, Fondazione IRCCS Istituto Nazionale dei Tumori di Milano, Milan, Italy; 5 Nutrition Therapy Unit, Fondazione IRCCS Istituto Nazionale dei Tumori di Milano, Milan, Italy; 6 Oncology and Hemato-oncology Department, Università degli Studi di Milano, Milan, Italy

**Keywords:** Cancer epidemiology, Tumour biomarkers

## Abstract

Previous works linked low sodium concentration with mortality risk in cancer. We aimed at weighing the prognostic impact of hyponatremia in all consecutive patients with metastatic solid tumors admitted in a two-years period at our medical oncology department. Patients were included in two cohorts based on serum sodium concentration on admission. A total of 1025 patients were included, of whom 279 (27.2%) were found to be hyponatremic. The highest prevalence of hyponatremia was observed in biliary tract (51%), prostate (45%) and small-cell lung cancer (38.9%). With a median follow-up of 26.9 months, median OS was 2 months and 13.2 months for the hyponatremia versus control cohort, respectively (HR, 2.65; *P* < 0.001). In the multivariable model, hyponatremia was independently associated with poorer OS (HR, 1.66; *P* < 0.001). According to the multivariable model, a nomogram system was developed and validated in an external set of patients. We weighed over time the influence of hyponatremia on survival of patients with metastatic solid tumors and pointed out the possibility to exploit serum sodium assessment to design integrated prognostic tools. Our study also highlights the need for a deeper characterization of the biological role of extracellular sodium levels in tumor development and progression.

## Introduction

Hyponatremia, as defined by a serum sodium concentration lower than 135 mEq/L, is the electrolyte alteration most frequently found in hospitalized patients, and a common finding at admission in Oncology Units^1,2^. In cancer patients, many overlapping factors can contribute to the onset of hyponatremia, including paraneoplastic syndromes (both cerebral salt wasting and syndrome of inappropriate antidiuretic hormone secretion, SIADH), clinical events (e.g. vomiting, diarrhea or bleeding) or syndromes (e.g., cachexia), anticancer treatments (cytotoxic agents, targeted therapies or immune checkpoint inhibitors) and general medications (e.g. opioids, diuretics and antidepressants)^3–7^. Albeit underdiagnosed, SIADH is one of the most common causes of euvolemic hyponatremia, especially in patients with small cell lung cancer (SCLC)^8^. Non-small cell lung cancer (NSCLC), breast cancer (BC), head and neck cancer (HNC), and colorectal cancer (CRC) are the non-hematologic malignancies most frequently associated with hyponatremia^3,8^. Regardless of the etiology, hyponatremia has been associated with high mortality risk both in cancer and non-cancer patients^9–12^, as well as with lower progression-free survival (PFS) and poorer response to treatments in patients with different malignancies^13–17^. Although previous studies showed an association between hyponatremia and poor patients’ survival in the metastatic setting^11,18–20^, the conclusions of these studies are intrinsically biased by small sample size, inclusion of patients with different tumor stages, or restriction to terminally ill patients. Here, we aimed at weighing the prognostic impact of hyponatremia in a large cohort of hospitalized patients with advanced solid tumors.

## Methods

### Patients’ population

Electronic registries of Fondazione IRCCS Istituto Nazionale dei Tumori of Milan were retrospectively searched in order to identify all patient admissions to the Medical Oncology department from January 1^st^ 2014 to December 31^st^ 2015. All admitted patients with advanced solid tumors were eligible and screened for serum sodium concentration on the admission day as part of a prospective departmental registry approved by the Institutional Review Board of Fondazione IRCCS Istituto Nazionale dei Tumori di Milano and according to the ethical principles for medical research involving human subjects adopted in the Declaration of Helsinki. All the participants signed an informed consent. Patients with at least one finding of hyponatremia at admission were included in the hyponatremia cohort. For patients presenting more than one hyponatremic event at admission during the study period, we only considered the hospitalization when hyponatremia was detected for the first time. Patients with no evidence of hyponatremia during any admission were included in the control cohort: in such cases, clinical and biological data regarding the first hospitalization occurring during the study period were recorded. Hyponatremia was classified as mild (130–134 mEq/L), moderate (125–129 mEq/L) or severe (<125 mEq/L) based on serum sodium concentration at admission, and in hypovolemic, hypervolemic or euvolemic based on the presence of causes of volume depletion or volume overload and based on the fluid status^21^. The following patient and tumor characteristics were collected: age, gender, ECOG PS, tumor histology, number and type of metastatic sites, number of previous lines of treatment, length of hospitalization and the presence of serum calcium or potassium alterations at admission. The following additional information was collected for hyponatremic patients: presence of factors contributing to the onset of hyponatremia, SIADH diagnosis and serum sodium concentration at discharge.

### Statistical analyses

Descriptive statistics were used to summarize baseline and hyponatremic patient characteristics, like counts and percentages for categorical variables, medians and interquartile ranges (IQRs) for continuous variables. We also assessed the frequency of hyponatremia among specific patient subgroups by means of Pearson’s chi square tests.

Overall survival (OS) time was calculated from the date of hospitalization to the date of death for any cause, with censoring for patients alive at the date of last follow up information. The Kaplan-Meier method was used for estimation of OS curves and related descriptive statistics, while the reverse Kaplan-Meier method described by Schemper and Smith^22^ was used for follow-up quantification. OS analysis relied on extensive use of Cox proportional hazards uni- and multivariable regression models according to a three-step strategy. Generalized boosted regression was used first for exploratory purposes, that is, to screen out irrelevant variables in terms of association with OS^23^. This tree-based regression approach, which is able to incorporate observations with partially missing data, also provided guidance for the detection of nonlinear effects and possible interactions among covariates, which was useful for the subsequent phase of analysis. As conventionally done, the variables with a relative influence lower than 1 were discarded, while the remaining variables were entered into a multivariable Cox proportional hazards regression model and selected with an Akaike Information Criterion (AIC)-based backward procedure^24^. The final step was addressed to fine-tune the model and visually describe the effect of prognostic factors through the use of nomograms. Cox model results were summarized using hazard ratios (HRs), together with the corresponding 95% confidence intervals (CI) and Wald’s p values, while model performance was assessed in terms of raw and bootstrap adjusted discrimination (Harrell’s c index)^25^. Nomogram external validation was performed on updated data from a cohort of 87 patients previously reported by Berardi *et al*.^19^. For this purpose, the validation series was stratified on the basis of nomogram predicted OS and stratum-specific Kaplan-Meier curves were estimated and compared with the logrank test. Furthermore, the overall agreement between observed and predicted deaths was assessed by means of a Poisson linear regression model. The modeling tools adopted were, respectively, Cox regression and linear models for normally distributed variables. Statistical analyses were carried out with SAS (version 9.4, SAS Institute, Cary, NC, USA) and R software (version 3.4.2, R Foundation for Statistical Computing, Vienna, Austria). Statistical significance was set at the conventional 5% two-sided threshold.

## Results

### Patients’ characteristics

Overall, 1025 patients hospitalized at our department from January 2014 to December 2015 were included, of whom 279 (27.2%) presented at least one finding of hyponatremia at admission during the study period. Supplementary Figure S1 and Supplementary Table S1 depict the CONSORT diagram of patients’ selection and their characteristics, respectively. The most represented histologies in our series were CRC (17.2%), NSCLC (17.0%), BC (16.5%) and gastroesophageal cancer (GEC) (8.5%). The highest prevalence of hyponatremia was observed in biliary tract cancer (BTC) (25 out of 49 patients, 51.0%), prostate cancer (PC) (9 out of 20 patients, 45.0%) and SCLC (14 out of 36 patients, 38.9%) (Table 1). Hyponatremia was positively associated with male gender, worse ECOG PS, higher number of metastatic sites, presence of liver, bone, brain, adrenal and lymph nodes metastases, higher number of previous treatment lines, and calcium or potassium imbalances (Table 1) while no association was observed between hyponatremia and age. Notably, patients in the hyponatremia cohort had a longer median length of hospitalization (8 days; IQR, 5 to 12 months) compared to patients in the control cohort (4 days; IQR, 3–8 months) (*P* < 0.001).

**Table 1:** Prevalence of hyponatremia according to patients’ characteristics.

Characteristics		*N*	%	95% CI	*P*
Gender					**<0.001**
	Male	160	32.3	28.2–36.6	
	Female	119	22.5	19–26.3	
Age (years)					0.3
	<65	158	28.5	24.8–32.4	
	≥65	121	25.7	21.9–30	
ECOG PS					**<0.001**
	0	33	12.1	8.5–16.6	
	1	100	22.7	18.9–26.9	
	2	51	49	39.1–59	
	3-4	93	60.4	52.2–68.2	
Histology					**<0.001**
	CRC	39	22.2	16.3–29	
	NSCLC	44	25.3	19–32.4	
	BC	35	20.7	14.9–27.6	
	GEC	30	34.5	24.6–45.4	
	Melanoma	19	32.2	20.6–45.6	
	PaC	18	34	21.5–48.3	
	BTC	25	51	36.3–65.6	
	RCC	11	28.2	15–44.9	
	SCLC	14	38.9	23.1–56.5	
	NET	7	25	10.7–44.9	
	PC	9	45	23.1–68.5	
	Other	28	20.7	14.3–28.6	
Metastatic sites (*N*)					**<0.001**
	1	62	19.2	15.1–23.9	
	2	77	24.8	20.1–29.9	
	≥3	140	35.8	31.1–40.8	
Liver metastases					**0.001**
	No	133	23.3	19.9–26.9	
	Yes	146	32.2	27.9–36.8	
Lung metastases					0.5
	No	175	26.6	23.2–30.1	
	Yes	104	28.4	23.9–33.3	
Bone metastases					**0.01**
	No	175	24.9	21.7–28.2	
	Yes	104	32.4	27.3–37.8	
Brain metastases					**0.02**
	No	238	26.1	23.3–29.1	
	Yes	41	36.3	27.5–45.9	
Adrenal metastases					**0.03**
	No	252	26.4	23.6–29.3	
	Yes	27	38	26.8–50.3	
Pleural metastases					0.1
	No	245	26.5	23.7–29.5	
	Yes	34	33.7	24.6–43.8	
Lymphnodal metastases					**<0.001**
	No	96	19	15.7–22.7	
	Yes	183	35.2	31.1–39.5	
Previous lines of treatment (*N*)					**<0.001**
	0	115	22.5	18.9–26.3	
	1–2	116	36.3	31–41.8	
	≥3	48	24.9	18.9–31.6	
Days of hospitalization					**<0.001**
	≤5	89	16.8	13.7–20.3	
	>5	190	38.4	34.1–42.8	
Other electrolyte imbalances					**<0.001**
	Calcium	42	35.6	27–44.9	
	Potassium	36	37.9	28.1–48.4	
	Both	22	40	27–54.1	
	None	179	24	21–27.2	

*Abbreviations*. IQR: interquartile range; NA: not available; CRC: colorectal cancer; NSCLC: non-small cell lung cancer; BC: breast cancer; GEC: gastroesophageal cancer; PaC: pancreatic cancer; BTC: biliary tract cancer; RCC: renal cell carcinoma; SCLC: small-cell lung cancer; NET: neuroendocrine tumor; PC: prostate cancer.

Specific characteristics of hyponatremic patients are shown in Table 2. Severe hyponatremia was detected in 12 (4.3%) out of 279 hyponatremic patients. An etiological diagnosis of hypovolemic (with coexisting volume depletion) or hypervolemic (with coexisting volume overload) hyponatremia was clearly established in 55 (19.7%) and 13 (4.7%) patients, respectively, while in the remaining 211 patients (75.6%) hyponatremia was considered to be euvolemic. Among patients with euvolemic hyponatremia, a diagnosis of SIADH was clearly established only in 5 (1.8% of the entire hyponatremia cohort). We found that 103 hyponatremic patients (36.9%) were receiving opioids when hyponatremia was detected, while concomitant use of diuretics and antidepressant drugs was observed in 29 (10.4%) and 20 (7.2%) patients, respectively. Specific treatments for hyponatremia consisted in fluid restriction in 79 patients (28.3%), administration of isotonic and hypertonic saline in 77 (27.6) and 118 patients (42.3%), respectively; finally, tolvaptan was prescribed to 5 (1.8%) patients with an established diagnosis of SIADH. A normalization in sodium level at discharge was observed in 113 patients (43.0%) with no differences in terms of normalization rate according to the type of hyponatremia (hypovolemic, hypervolemic or euvolemic) (*P* = 0.23). 44 patients (15.8%) were readmitted with recurrence or persistence of hyponatremia during the study period. Normalization in sodium level at discharge was negatively associated with the chance of recurrence or persistence of hyponatremia (odds ratio, 0.36; 95% CI, 0.17–0.76; P = 0.008).

**Table 2:** Specific patients’ characteristics for the hyponatremia cohort.

Characteristics	Hyponatremia cohort (*N* = 279) *N* (%)	
Sodium level at admission (mEq/l)		
	Median	133
	IQR	131–134
Hyponatremia grade		
	Moderate	231 (82.8)
	Mild	36 (12.9)
	Severe	12 (4.3)
Type of hyponatremia		
	Hypovolemic	55 (19.7)
	Hypervolemic	13 (4.7)
	Euvolemic	211 (75.6)
Normal sodium level at discharge		
	No	150 (57.0)
	Yes	113 (43.0)
	NA	16
Persistance of hyponatremia at further admissions		
	No	235 (84.2)
	Yes	44 (15.8)

*Abbreviations*. IQR: interquartile range; SIADH: syndrome of inappropriate antidiuretic hormone secretion; NA: not available.

### Impact of hyponatremia on overall survival

**Figure 1:**
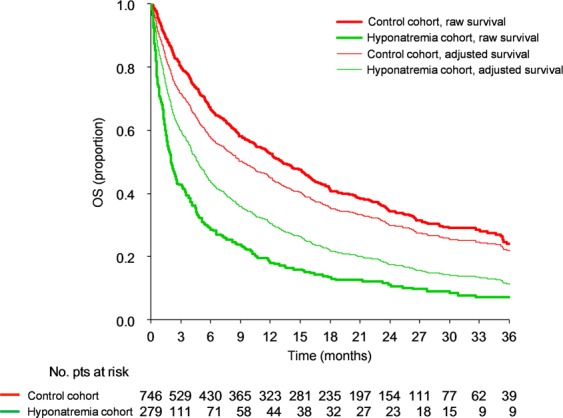
Kaplan-Meier curves for overall survival. Red lines indicate patients in the control cohort while green lines indicate patients in the hyponatremia cohort. Thick lines indicate raw survival curves while thin lines indicate adjusted survival curves. Patients in the control cohort had higher overall survival compared to patients in the hyponatremia cohort.

**Figure 2:**
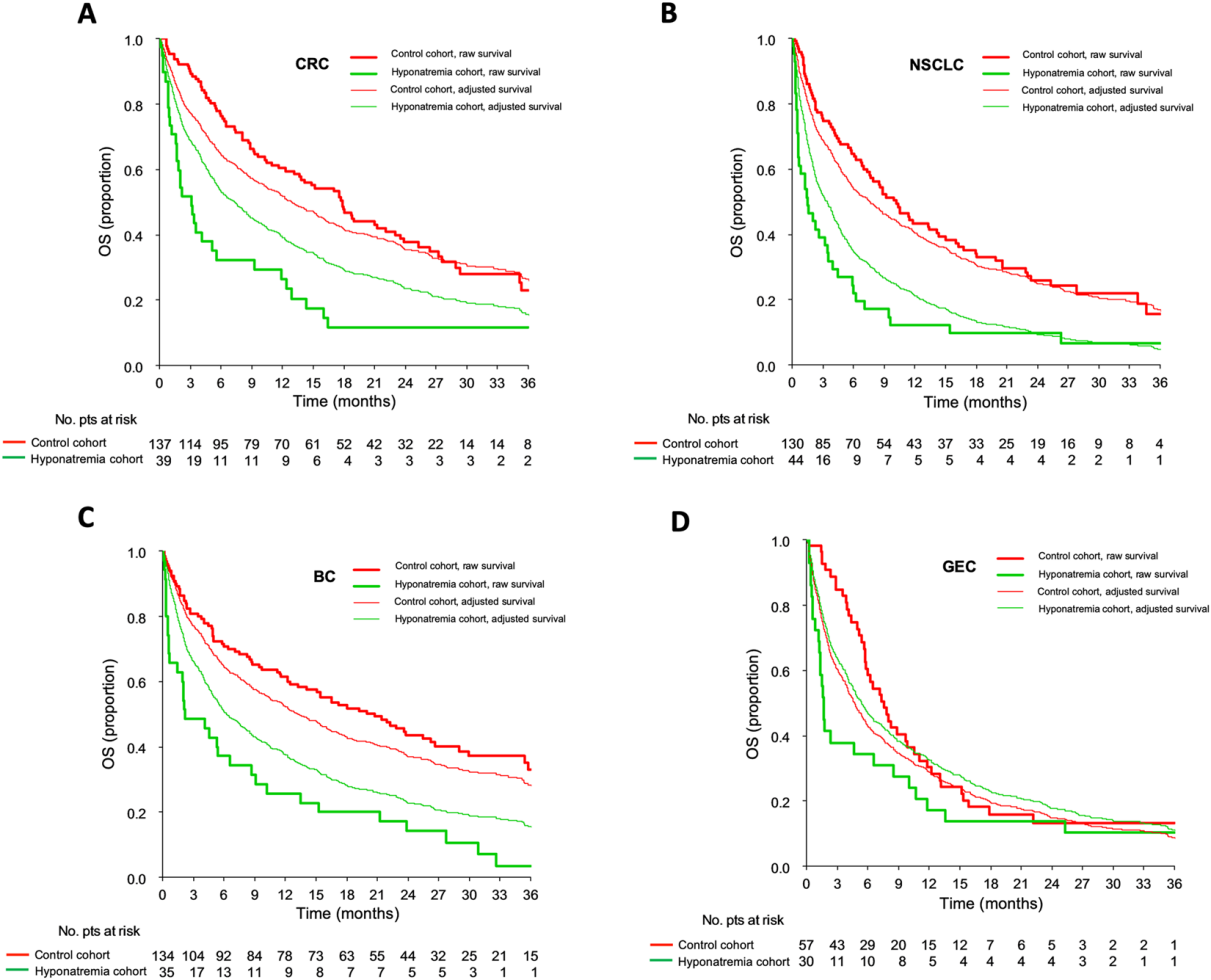
Kaplan-Meier curves for overall survival regarding the four most represented histologies. Red lines indicate patients in the control cohort while green lines indicate patients in the hyponatremia cohort. Thick lines indicate raw survival curves while thin lines indicate adjusted survival curves. Patients in the control cohort had higher overall survival compared to patients in the hyponatremia cohort in colorectal cancer (CRC, panel A), non-small cell lung cancer (NSCLC, panel B) and breast cancer (BC, panel C). In gastroesophageal cancer (GEC, panel D), adjusted model indicates that patients in the hyponatremia cohort had a better prognosis than patients in the control cohort.

With a median follow-up of 26.9 months (IQR, 19.8–35.6 months), death for all causes was recorded in 241 (86.4%) patients in the hyponatremia cohort and 450 (60.3%) in the control cohort. Median overall survival (OS) was 2 months (IQR, 0.7–8.2 months) and 13.2 months (IQR, 4.1–35.4 months) for hyponatremic versus control patients, respectively (HR, 2.65; 95% CI, 2.26–3.11; *P* < 0.001) (Fig. 1 and Supplementary Table S2). Survival curves and HRs of patients with the four most represented tumor histologies in our study population are shown in Fig. 2 and Supplementary Table S2. When the weight of each variable on overall survival was assessed, ECOG PS and histology showed the greatest relative influence (Supplementary Table S3). In the multivariable model (Table 3), all covariates (age, ECOG PS, histology, presence of liver, bone and brain metastases, number of completed lines of treatment and other electrolyte imbalances) were significantly associated with OS. Of note, hyponatremia was associated with poorer OS independently from other variables (HR, 1.66; 95% CI, 1.38–2.01; *P* < 0.001). Accordingly, adjusted median OS for the hyponatremia versus control cohort was 4.7 months (IQR, 1.5–15.6 months) and 9.2 months (IQR, 2.4–32.6 months) (Fig. 1). Adjusted survival curves and HRs for the four most represented histologies are shown in Fig. 2 and Supplementary Table S2.

**Table 3:** Multivariable proportional hazard regression model on overall survival.

Characteristic		HR	95% CI	*P*
Cohort				**<0.001**
	Hyponatremia vs Control	1.66	1.38–2.01	
Age (years)				**<0.001**
	71 vs 53^a^	1.36	1.20–1.55	
ECOG PS				**<0.001**
	1 vs 0	1.31	1.06–1.61	<0.01
	2 vs 0	1.8	1.32–2.44	<0.001
	3 vs 0	2.91	2.16–3.90	<0.001
	4 vs 0	8.62	4.40–16.88	0.001
Histology				**<0.001**
	NSCLC vs CRC	1.4	1.05–1.88	0.02
	BC vs CRC	0.85	0.64–1.14	0.3
	GEC vs CRC	1.75	1.27–2.39	<0.001
	Melanoma vs CRC	1.52	1.04–2.22	0.03
	PaC vs CRC	2.42	1.65–3.55	<0.001
	BTC vs CRC	2.69	1.82–3.99	<0.001
	RCC vs CRC	0.3	0.16–0.56	0.001
	SCLC vs CRC	1.61	1.02–2.53	0.04
	NET vs CRC	0.97	0.56–1.66	0.9
	PC vs CRC	0.67	0.36–1.23	0.2
	Other vs CRC	1.08	0.79–1.47	0.6
Liver metastases				**<0.001**
	Yes vs No	1.7	1.43–2.02	
Bone metastases				**<0.001**
	Yes vs No	1.39	1.16–1.67	
Brain metastases				**<0.001**
	Yes vs No	1.74	1.32–2.28	
Previous lines of treatment (*N*)				**<0.001**
	1–2 vs 0	1.39	1.16–1.67	<0.001
	>2 vs 0	1.59	1.26–2.02	<0.001
Days of hospitalization				**0.005**
	9 vs 3^a^	1.13	1.04–1.23	
Other electrolyte imbalances				**0.04**
	Calcium vs None	1.32	1.03–1.68	0.03
	Potassium vs None	0.87	0.66–1.14	0.3
	Both vs None	1.25	0.91–1.71	0.2
Harrell *c*-*index*				
	Apparent: 0.75			
	Bias-corrected: 0.74			

*Abbreviations*. HR: hazard ratio; CRC: colorectal cancer; NSCLC: non-small cell lung cancer; BC: breast cancer; GEC: gastroesophageal cancer; PaC: pancreatic cancer; BTC: biliary tract cancer; RCC: renal cell carcinoma; SCLC: small-cell lung cancer; NET: neuroendocrine tumor; PC: prostate cancer.

^a^The two values are, respectively, the 3rd and 1st quartiles of the variable distribution.

Results of the Cox proportional hazards regression model when testing the role of specific covariates in the hyponatremia cohort are shown in Supplementary Table S4. In particular, only the grade of hyponatremia was associated with survival (*P* = 0.02), with an incremental risk for death for mild (HR, 1.61; 95% CI, 1.32–1.97), moderate (HR, 1.87; 95% CI, 1.25–2.78) and profound hyponatremia (HR, 2.29; 95% CI, 1.17–4.50) (Supplementary Figure S2). We found no survival differences in patients with normalized or not normalized serum sodium levels at discharge (Supplementary Table S4).

### Development and validation of a nomogram including hyponatremia

In order to exploit the prognostic role of hyponatremia for a better risk-stratification of patients with advanced solid tumors, we developed a nomogram scoring system to predict the early (3-months) and late (36-months) OS probabilities.

**Figure 3:**
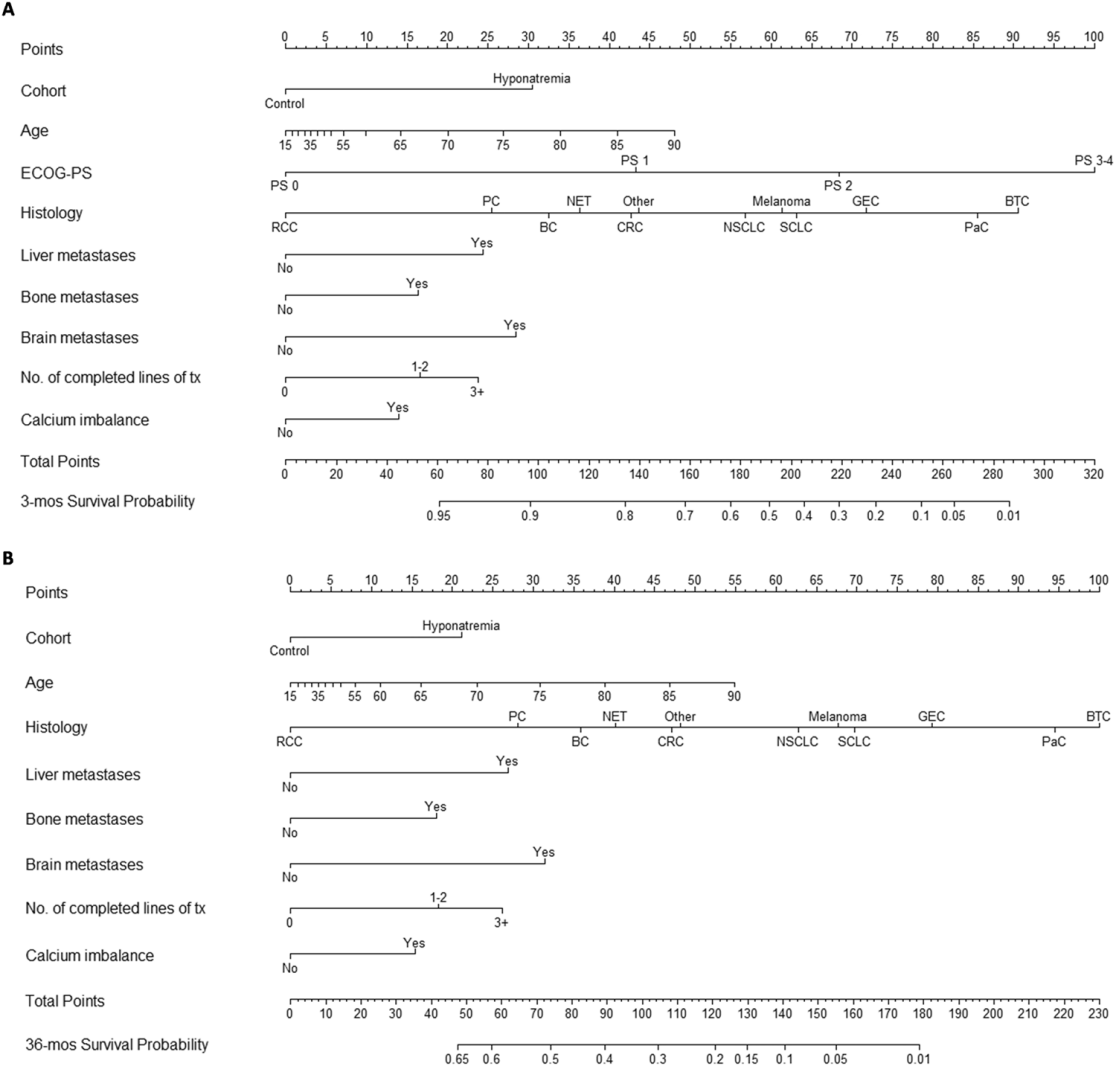
Nomograms predicting the survival probability within 3 months (panel A) and 36 months (panel B) after the date of hospitalization. Abbreviations. BC: breast cancer, BTC: biliary tract cancer, CRC: colorectal cancer, GEC: gastroesophageal cancer, mos: months, NET: neuroendocrine tumor, NSCLC: non-small cell lung cancer, PaC: pancreatic cancer, PC: prostate cancer, RCC: renal cell carcinoma, SCLC: small-cell lung cancer, tx: therapy.

According to the multivariable model, the presence of hyponatremia, age, ECOG PS, histology, liver, bone and brain metastases, number of previous lines of treatment, and the presence of calcium imbalances were used to develop the nomograms. The proportional hazards assumption was violated for hyponatremia and ECOG PS in a way that we could address by means of time stratification. The nomograms are shown in Fig. 3 and predict the probability that patients will be alive at 3 months and 36 months after the date of hospitalization. Supplementary Data S1 reports the model equations, which can be used for a more precise calculation of predictions. Patients characteristics of the validation cohort are reported in Supplementary Table S5. When applied to the validation set, our nomogram scoring system confirmed its discriminative ability for prognostic stratification (Supplementary Figure S3), even though the observed number of deaths exceeded the number of nomogram predicted deaths by around 50% (Ratio, 1.56; 95% CI, 1.22–1.98).

## Discussion

Weighing the impact of hyponatremia on survival of patients with advanced solid tumors could improve their prognostic stratification and facilitate the process of treatment decision making. In this retrospective study, we investigated the prognostic impact of hyponatremia in a heterogeneous population of hospitalized patients with metastatic or relapsed solid tumors admitted to a tertiary medical oncology department in a two-years period.

We observed a prevalence of hyponatremia at admission in hospitalized metastatic cancer patients of 27.2%. Since we did not take into account hyponatremia emerging during patient hospitalization, nor hyponatremia occurring in out-patients or in non-metastatic patients, the prevalence of hyponatremia in our study was lower than reported in previous retrospective studies (38–63.7%)^2,18–20^. Hyponatremia was clearly associated with tumor histology, being more frequent in small cell lung cancer, prostate cancer and biliary tract cancer. In particular, the high prevalence of hyponatremia in biliary tract cancer is consistent with the incidence of this electrolyte alteration reported in clinical trials with different chemotherapy combinations^26–29^, suggesting that treatment-related adverse events might contribute to the high prevalence of hyponatremia in biliary tract cancer. Noteworthy, hyponatremic patients had worse ECOG PS, higher prevalence of other electrolyte imbalances and higher number of metastatic sites or disease burden, suggesting more compromised general status^18^. In our study, patients with hyponatremia had a significantly lower OS, with a median time of only 2 months versus 13.2 months observed in the control cohort. Hyponatremia also retained an independent negative prognostic role in the multivariable model (HR, 1.66; 95% CI, 1.38–2.01; *P* < 0.001), in line with the available literature^6,19^. A subgroup analysis for the four most prevalent tumor histologies confirmed the negative prognostic impact of hyponatremia for colorectal cancer, non-small cell lung cancer and breast cancer^15,16,18^, but not for gastroesophageal cancer, a tumor type for which evidence from the literature is scarce and conflicting^30,31^. Notably, we observed an incremental death risk when stepping from mild to moderate and severe hyponatremia, thus confirming previously published data^11,19,20^ (Supplementary Figure S2, panel A).

Even if hyponatremia was reported to influence OS mainly within 6 months, it also retained its prognostic role at later time points, as previously shown in colorectal cancer^16^. Moreover, in our externally-validated nomogram scoring system, hyponatremia showed a remarkable weight both on 3-months and 36-months OS probabilities. Especially, in our 36-months model, ECOG PS failed to show a relevant influence on survival and was not included in the nomogram. Remarkably, our nomogram scoring system could be easily used to facilitate the process of treatment decision making based on life-expectancy of heavily-pretreated cancer patients (e.g. inclusion in clinical studies vs best supportive care). Regarding the higher number of observed death events relative to the number of nomogram predicted deaths in the validation cohort, it might be probably due to referral patterns. Although hyponatremia has been associated with critical conditions^32^, our findings indicate that hyponatremia may worsen the prognosis of metastatic cancer patients independently from patients’ general status. Interestingly, hyponatremia has been associated with worse PFS and lower response to treatment of cancer patients^13,15,17,18,33^, thus suggesting a possible, direct impact on cancer progression and/or resistance to treatments. For example, even if transient, hyponatremia activates the rennin–angiotensin–aldosterone system that is implicated in malignant transformation and cancer cell survival^34–37^. Hyponatremia could also interfere with the regulation and activity of sodium channels and sodium-involving ion pumps (e.g. voltage-gated sodium channels and epithelial sodium channel), whose aberrant expression has been found in multiple cancer types, directly contributing to cancer development and progression^38–41^. Furthermore, low sodium levels could stabilize or enhance via hypotonic stress the transcription of the glucocorticoid-induced protein kinase 1 (SGK1), which has been recently linked to cancer cell metastatization^42,43^.

Taken together and corroborated by our findings, these data imply that hyponatremia could have a pleiotropic action in cancer promotion and progression, thus explaining its unfavorable prognostic role in metastatic cancer patients beyond the traditional association with poorer patient performance status or tumor burden. Our study has some limitations: 1) it is a retrospective study (even if it relies on a prospective departmental registry) and we believe that a prospective study could be the ideal strategy to definitely validate the prognostic role of hyponatremia in patients with advanced malignancies; 2) we only included hospitalized patients, while extending a comprehensive assessment of the prognostic role of hyponatremia also in an out-patient setting would improve the solidity and reproducibility of our findings; 3) despite an external validation, our nomogram scoring system needs to be further tested for non–hospitalized metastatic cancer patients.

In conclusion, our study clearly confirmed the prognostic role of hyponatremia and weighed its impact on the OS of hospitalized patients with metastatic solid tumors. Based on our findings, serum sodium assessment could be exploited to design integrated prognostic tools, at least for hospitalized patients. A strong clinical and translational effort is needed to characterize the biological role of extracellular sodium levels and the interplay with ion channels located on tumor cell membranes in promoting cancer initiation, progression and resistance to treatment.

## Supplementary information

Supplementary Information

## Data Availability

The datasets generated during and/or analyzed during the current study are available from the corresponding author on reasonable request.
