# Supplementary material for: Weighing the prognostic role of hyponatremia in hospitalized patients with metastatic solid tumors: the HYPNOSIS study
Source: Sci Rep. 2019 Sep 10;9:12993. doi: 10.1038/s41598-019-49601-3 (PMC6736887; doi:10.1038/s41598-019-49601-3)
Supplement: Supplementary file 1 — Supplementary Information [file 41598_2019_49601_MOESM1_ESM.pdf]

Weighing the prognostic role of hyponatremia in hospitalized patients with metastatic solid tumors: the HYPNOSIS study.

Giovanni Fucà, Luigi Mariani, Salvatore Lo Vullo, Giulia Galli, Rossana Berardi, Massimo Di Nicola, Claudio Vernieri, Daniele Morelli, Katia Dotti, Ilaria Fiordoliva, Silvia Rinaldi, Cecilia Gavazzi, Filippo Pietrantonio, Marco Platania & Filippo de Braud

Supplementary Information

Supplementary Table S1

Supplementary Table S2

Supplementary Table S3

Supplementary Table S4

Supplementary Table S5

Supplementary Figure S1

Supplementary Figure S2

Supplementary Figure S3

Supplementary Data S1

Supplementary Table S1. Patients' characteristics for the entire series.

		Entire series (N=1025)	
**Characteristics**		***N***	**%**
**Cohort**			
	Control	746	72.8
	Hyponatremia	279	27.2
**Gender**			
	Male	495	48.3
	Female	530	51.7
**Age** (years)			
	Median	63	
	IQR	53-71	
**ECOG PS**			
	0	272	28.0
	1	440	45.4
	2	104	10.7
	3-4	154	15.9
	NA	55	-
**Histology**			
	CRC	176	17.2
	NSCLC	174	17.0
	BC	169	16.5
	GEC	87	8.5
	Melanoma	59	5.8
	PaC	53	5.2
	BTC	49	4.8
	RCC	39	3.8
	SCLC	36	3.5
	NET	28	2.7
	PC	20	2.0
	Other	135	13.2
**Metastatic sites** (*N*)			
	1	323	31.5
	2	311	30.3
	≥ 3	391	38.1
**Liver metastases**			
	No	572	55.8
	Yes	453	44.2
**Lung metastases**			
	No	659	64.3
	Yes	366	35.7
**Bone metastases**			
	No	704	68.7
	Yes	321	31.3
**Brain metastases**			
	No	912	89.0
	Yes	113	11.0
**Adrenal metastases**			
	No	954	93.1
	Yes	71	6.9
**Pleural metastases**			
	No	924	90.1

	Yes	101	9.9
**Lymphnodal metastases**			
	No	505	49.3
	Yes	520	50.7
**Completed lines of treatment** (*N*)			
	0	512	50.0
	1-2	320	31.2
	≥ 3	193	18.8
**Days of hospitalization**			
	Median	5	
	IQR	3-9	
**Other electrolite imbalances**			
	Calcium	118	11.6
	Potassium	95	9.4
	Both	55	5.4
	None	746	73.6
	NA	11	-

Abbreviations. IQR: interquartile range; NA: not available; CRC: colorectal cancer; NSCLC: non-small cell lung cancer; BC: breast cancer; GEC: gastroesophageal cancer; PaC: pancreatic cancer; BTC: biliary tract cancer; RCC: renal cell carcinoma; SCLC: small-cell lung cancer; NET: neuroendocrine tumor; PC: prostate cancer.

Supplementary Table S2. Raw and adjusted overall survival estimates, according to cohort and main histologies.

		Raw			Adjusted		
Histology	Cohort	Median (IQR)	HR (95% CI)	*P*	Median (IQR)	HR (95% CI)	*P*
**Any**	Hyponatremia	2.0 (0.7 - 8.6)	2.65 (2.26 - 3.11)	**< .001**	4.7 (1.5 - 15.6)	1.66 (1.38 - 2.01)	**< .001**
	Control	13.2 (4.1 - 35.4)			9.2 (2.4 - 32.6)		
**CRC**	Hyponatremia	3.1 (1.0 - 12.4)	2.94 (1.96 - 4.41)	**< .001**	7.3 (2.1 - 23.3)	1.52 (1.00 - 2.32)	.05
	Control	17.9 (6.3 - 35.3)			12.9 (3.7 - 36.3)		
**NSCLC**	Hyponatremia	1.5 (0.5 - 5.9)	2.65 (1.80 - 3.90)	**< .001**	3.4 (1.2 - 10.0)	1.97 (1.31 - 2.95)	**.001**
	Control	10.2 (3.0 - 25.3)			7.6 (2.1 - 24.0)		
**BC**	Hyponatremia	2.1 (0.5 - 13.4)	2.70 (1.80 - 4.06)	**< .001**	6.3 (1.9 - 22.8)	1.70 (1.10 - 2.61)	**.02**
	Control	20.7 (4.9 - 38.8)			13.2 (3.6 - 38.0)		
**GEC**	Hyponatremia	1.6 (0.9 - 10.1)	1.72 (1.06 - 2.80)	**.03**	5.4 (1.8 - 16.4)	0.88 (0.53 - 1.47)	.63
	Control	7.6 (4.4 - 13.1)			4.7 (1.5 - 14.2)		

Abbreviations. OS: overall survival; IQR: interquartile range; CRC: colorectal cancer; NSCLC: non-small cell lung cancer; BC: breast cancer; GEC: gastroesophageal cancer.

Supplementary Table S3. Screening of the association between patients' characteristics and overall survival by means of generalized boosted regression.

Characteristic	Relative influence
ECOG PS	30.89031746
Histology	27.72414656
Metastatic sites (*N*)	9.78995113
Age	9.69024764
Liver metastases	6.68089315
Completed lines of treatment (*N*)	5.17719910
Other electrolyte imbalances	4.41920052
Brain metastases	1.55851726
Bone metastases	1.47475542
Gender	1.12306497
Lymphnodal metastases	0.30934482
Lung metastases	0.13015533
Pleural metastases	0.11742047
Adrenal metastases	0.11098979

Supplementary Table S4. Cox proportional hazards regression model including specific covariates in the hyponatremia cohort.

Characteristic		HR	95% CI	*P*
**Hyponatremia grade**				**.02**
	Mild	1.61	1.32-1.97	
	Moderate	1.87	1.25-2.78	
	Profound	2.29	1.17-4.50	
**SIADH diagnosis**				.55
	No	1.66	1.38-2.01	
	Yes	1.76	0.55-5.71	
**Persistance of hyponatremia at further admissions**				.08
	No	1.66	1.36-2.02	
	Yes	1.71	1.20-2.43	
**Normal sodium level at discharge**				.13
	No	1.99	1.57-2.53	
	Yes	1.46	1.15-1.87	

Abbreviations. HR: hazard ratio; CI: confidence interval; SIADH: syndrome of inappropriate antidiuretic hormone secretion

Supplementary Table S5. Patients' characteristics of the validation set.

		Entire series (*N*= 87)	
**Characteristics**		**N**	**%**
**Cohort**			
	Control	68	78.2
	Hyponatremia	19	21.8
**Sex**			
	Male	52	59.8
	Female	35	40.2
**Age** (years)			
	Median	67	
	IQR	55-74	
**ECOG PS**			
	0	23	26.4
	1	24	27.6
	2	26	29.9
	3-4	14	16.1
**Histology**			
	CRC	16	18.4
	NSCLC	29	33.3
	BC	6	6.9
	GEC	4	4.6
	Melanoma	2	2.3
	PaC	2	2.3
	BTC	1	1.1
	RCC	4	4.6
	SCLC	-	-
	NET	3	3.4
	PC	2	2.3
	Other	18	20.7
**Metastatic sites** (*N*)			
	1	20	23.0
	2	35	40.2
	≥ 3	32	36.8
**Liver metastases**			
	No	49	56.3
	Yes	38	43.7
**Lung metastases**			
	No	59	67.8
	Yes	28	32.2
**Bone metastases**			
	No	63	72.4
	Yes	24	27.6
**Brain metastases**			
	No	73	83.9
	Yes	14	16.1
**Adrenal metastases**			
	No	77	88.5
	Yes	10	11.5
**Pleural metastases**			
	No	69	79.3
	Yes	18	20.7
**Lymphnodal metastases**			

	No	39	44.8
	Yes	48	55.2
**Completed lines of treatment** (*N*)			
	0	40	46.0
	1-2	41	47.1
	≥ 3	6	6.9
**Days of hospitalization**			
	Median	9	
	IQR	5-17	
**Other electrolyte imbalances**			
	Calcium	10	11.5
	Potassium	6	6.9
	Both	-	-
	None	71	81.6

Abbreviations. IQR: interquartile range; NA: not available; CRC: colorectal cancer; NSCLC: non-small cell lung cancer; BC: breast cancer; GEC: gastroesophageal cancer; PaC: pancreatic cancer; BTC: biliary tract cancer; RCC: renal cell carcinoma; SCLC: small-cell lung cancer; NET: neuroendocrine tumor; PC: prostate cancer.

Supplementary Figure S1. CONSORT diagram depicting the process of patients' selection. Briefly, admissions were screened for serum sodium concentration on the admission day. Patients with at least one finding of hyponatremia at hospitalization were considered for inclusion in the hyponatremia cohort while patients with no evidence of hyponatremia at any admission were considered for inclusion in the control cohort. Only patients with metastatic or relapsed solid tumors were finally included in the two cohorts.

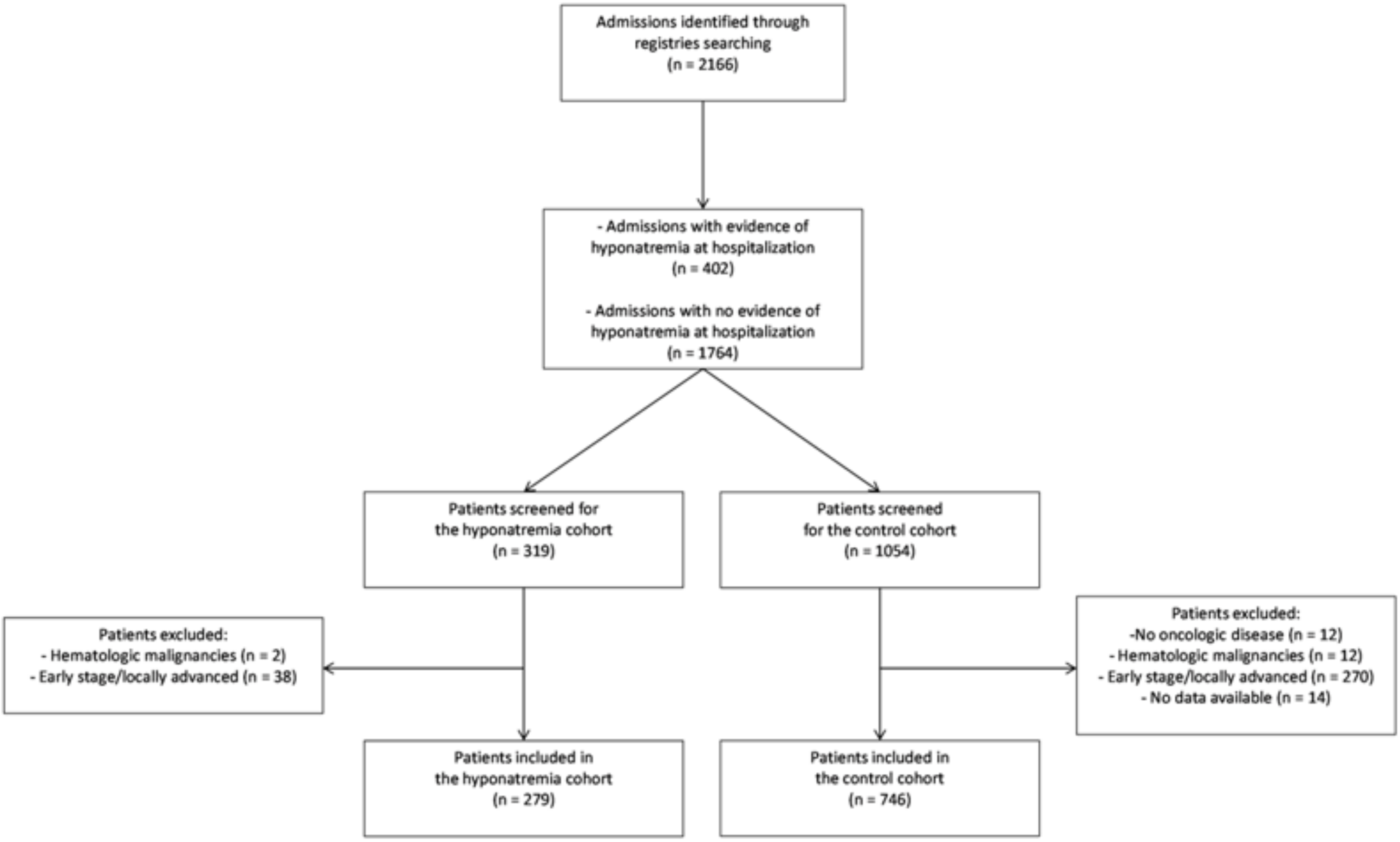

Supplementary Figure S2. Kaplan-Meier curves for overall survival according to the grade of hyponatremia. An incremental risk for death was observed for patients in the control cohort (red line) compared with patients with mild (green line), moderate (dark blue) and profound hyponatremia (light blue).

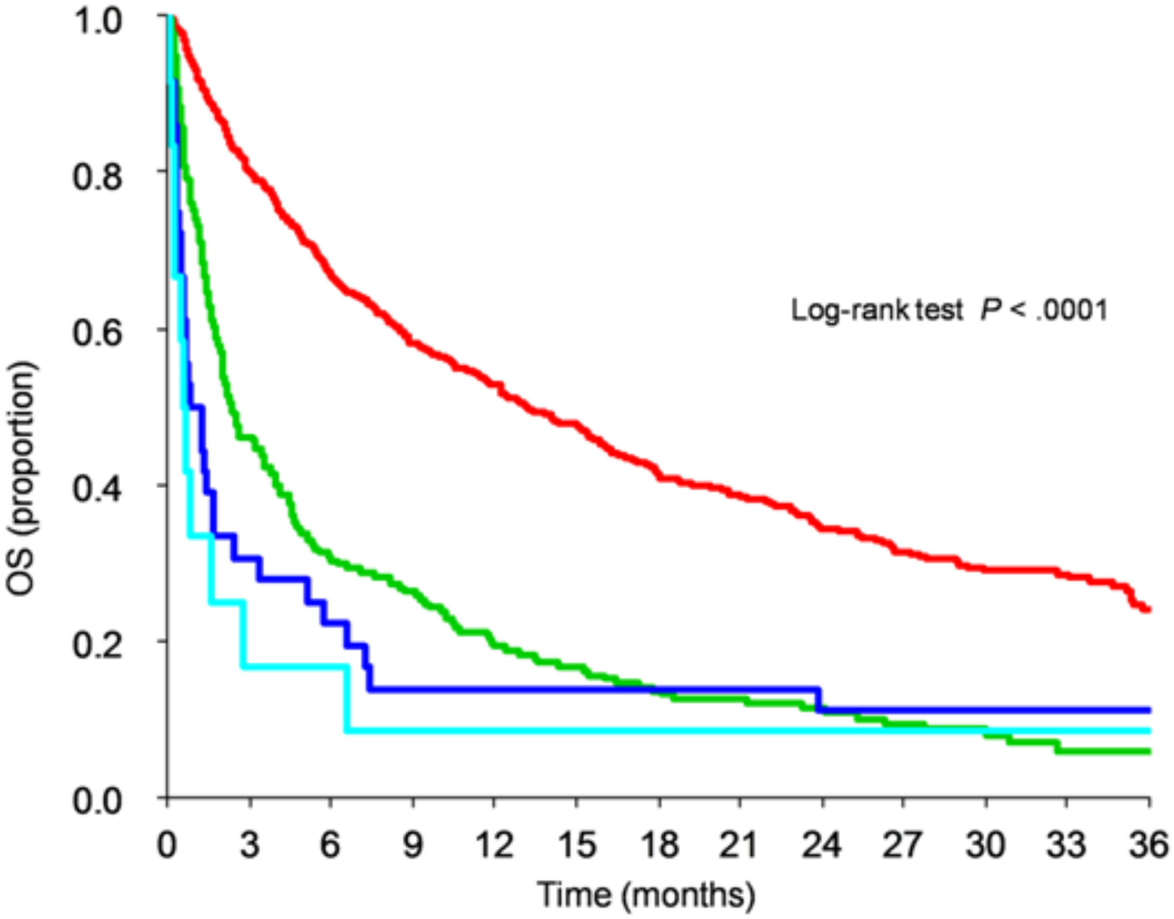

No. pts at risk

Control	746	529	430	365	323	281	235	197	154	111	77	62	39
Mild	231	98	61	52	38	32	26	21	18	14	11	5	5
Moderate	36	11	8	5	5	5	5	5	4	3	3	3	3
Profound	12	2	2	1	1	1	1	1	1	1	1	1	1

Supplementary Figure S3. Kaplan-Meier curves for overall survival in the validation series according to the tertiles of nomogram predicted probabilities. *P* value of the logrank test performed to compare the curves, confirmed the efficacy in achieving prognostic stratification of our nomogram scoring system for patients in the first (red line), second (green line) and third (blue) tertiles.

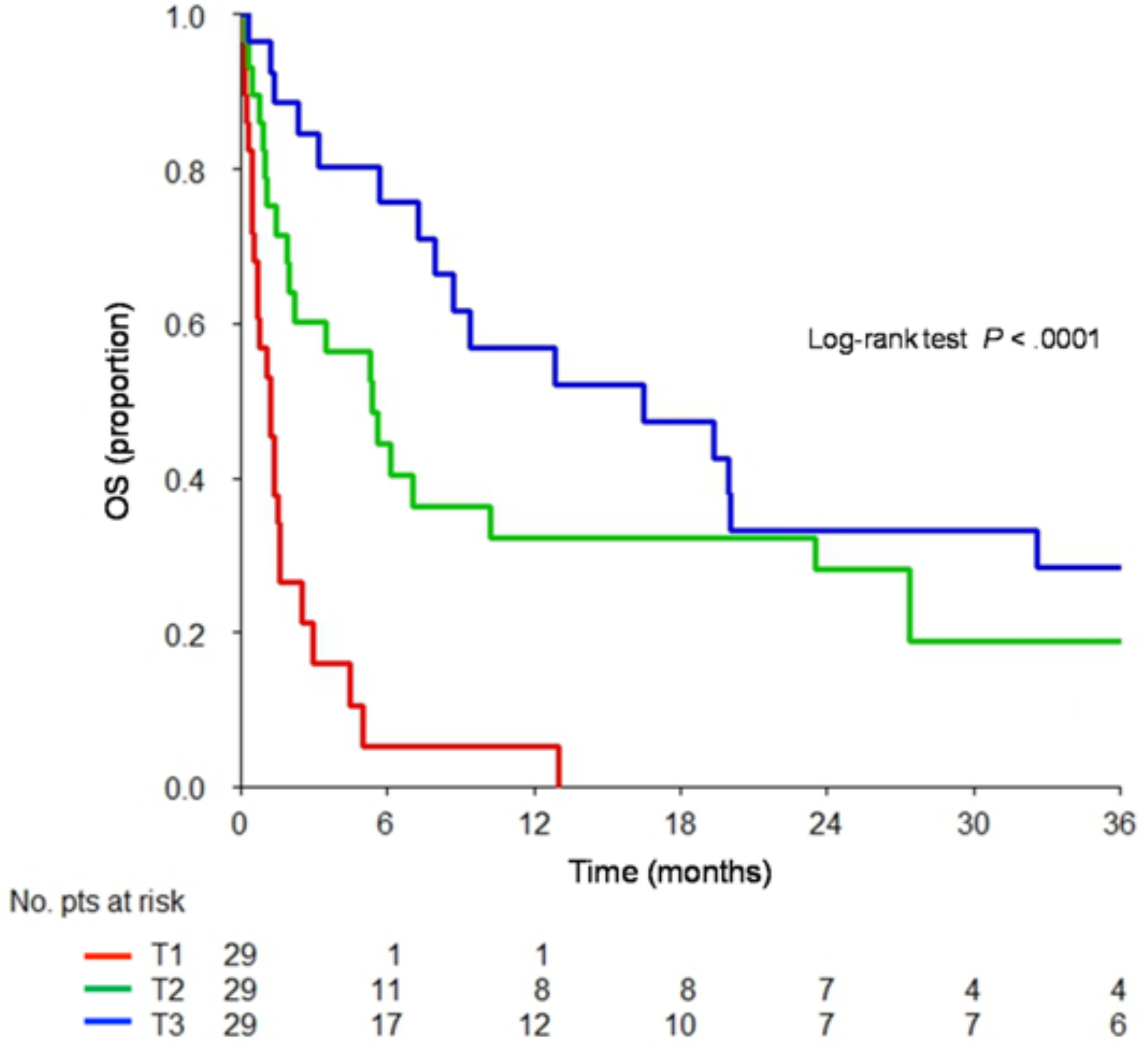

Supplementary Data S1. Model equations

Generic equation:

$$S_0^{\exp(LP)}$$

Baseline survival estimates S_0 according to model and time.

Model	Time	
	3 months	36 months
#1 (*1st period*)	0.822717	-
#2 (*2nd period*)	-	0.097244

For model #1 (*1st period*), LP= *a*

where *a* = - 1.74338 + I[cohort=Hyponatremia] × 0.60683 + Age × 0.00312 + Age' × 0.01403
+ I[ECOG-PS=PS 1] × 0.86341 + I[ECOG-PS=PS 2] × 1.36425
+ I[ECOG-PS=PS 3-4] × 1.99461 + I[Histology=NSCLC] × 0.28299
- I[Histology=BC] × 0.20273 + I[Histology=GEC] × 0.58011
+ I[Histology=Melanoma] × 0.37174 + I[Histology=PaC] × 0.85372
+ I[Histology=BTC] × 0.95403 - I[Histology=RCC] × 0.85100
+ I[Histology=SCLC] × 0.40761 - I[Histology=NET] × 0.12678 - I[Histology=PC] × 0.34323
+ I[Histology=Other] × 0.01981 + I[Liver metastases=Yes] × 0.48510
+ I[Bone metastases=Yes] × 0.32575 + I[Brain metastases=Yes] × 0.56768
+ I[No. of completed lines of tx=1-2] × 0.33069 + I[No. of completed lines of tx=3+] × 0.47320
+ I[Calcium imbalance=Yes] × 0.27744 .

For model #2 (*2nd period*), LP= *b*

where *b* = - 1.74338 + I[cohort=Hyponatremia] × 0.38241 + Age × 0.00312 + Age' × 0.01403
+ I[Histology=NSCLC] × 0.28299 - I[Histology=BC] × 0.20273
+ I[Histology=GEC] × 0.58011 + I[Histology=Melanoma] × 0.37174
+ I[Histology=PaC] × 0.85372 + I[Histology=BTC] × 0.95403 - I[Histology=RCC] × 0.85100
+ I[Histology=SCLC] × 0.40761 - I[Histology=NET] × 0.12678 - I[Histology=PC] × 0.34323
+ I[Histology=Other] × 0.01981 + I[Liver metastases=Yes] × 0.48510
+ I[Bone metastases=Yes] × 0.32575 + I[Brain metastases=Yes] × 0.56768
+ I[No. of completed lines of tx=1-2] × 0.33069 + I[No. of completed lines of tx=3+] × 0.47320
+ I[Calcium imbalance=Yes] × 0.27744 .

In both *a* and *b*,

$$Age' = \left[(Age-44)_+^{\,3} - (Age-63)_+^{\,3} \times \frac{(76-44)}{(76-63)} + (Age-76)_+^{\,3} \times \frac{(63-44)}{(76-63)}\right] / (76-44)^2$$

$$\cong$$

$$\cong \left[(Age-44)_+^{\,3} - (Age-63)_+^{\,3} \times 2.46154 + (Age-76)_+^{\,3} \times 1.46154\right] / 1024\,,$$

being 44, 63, 76 the values corresponding to the 10th, 50th, 90th percentiles, respectively, of the distribution of *Age.* (See equations 2.25 and 2.26 in: *Harrell FJ Jr. Regression modeling strategies: with applications to linear models, logistic regression, and survival analysis, 2nd ed. Springer series in statistics. New York: Springer-Verlag, 2001.*)

Notes: The indicator function I[*condition*] is 1 when the *condition* is satisfied, or 0 otherwise.
The notation $(X-c)_+$ is intended to take the value $(X-c)$ itself when it's positive, or 0 otherwise.
